# Def1 Promotes the Degradation of Pol3 for Polymerase Exchange to Occur During DNA-Damage–Induced Mutagenesis in *Saccharomyces cerevisiae*

**DOI:** 10.1371/journal.pbio.1001771

**Published:** 2014-01-21

**Authors:** Andreea Daraba, Vamsi K. Gali, Miklós Halmai, Lajos Haracska, Ildiko Unk

**Affiliations:** The Institute of Genetics, Biological Research Centre, Hungarian Academy of Sciences, Szeged, Hungary; Max Planck Institute of Biochemistry, Germany

## Abstract

DNA damages hinder the advance of replication forks because of the inability of the replicative polymerases to synthesize across most DNA lesions. Because stalled replication forks are prone to undergo DNA breakage and recombination that can lead to chromosomal rearrangements and cell death, cells possess different mechanisms to ensure the continuity of replication on damaged templates. Specialized, translesion synthesis (TLS) polymerases can take over synthesis at DNA damage sites. TLS polymerases synthesize DNA with a high error rate and are responsible for damage-induced mutagenesis, so their activity must be strictly regulated. However, the mechanism that allows their replacement of the replicative polymerase is unknown. Here, using protein complex purification and yeast genetic tools, we identify Def1 as a key factor for damage-induced mutagenesis in yeast. In *in vivo* experiments we demonstrate that upon DNA damage, Def1 promotes the ubiquitylation and subsequent proteasomal degradation of Pol3, the catalytic subunit of the replicative polymerase δ, whereas Pol31 and Pol32, the other two subunits of polymerase δ, are not affected. We also show that purified Pol31 and Pol32 can form a complex with the TLS polymerase Rev1. Our results imply that TLS polymerases carry out DNA lesion bypass only after the Def1-assisted removal of Pol3 from the stalled replication fork.

## Abstract 2

After DNA damage, Def1 triggers degradation of the catalytic subunit of the replicative DNA polymerase at stalled replication forks, allowing special polymerases to take over DNA synthesis.

## Author Summary

DNA damages can lead to the stalling of the cellular replication machinery if not repaired on time, inducing DNA strand breaks, recombination that can result in gross chromosomal rearrangements, even cell death. In order to guard against this outcome, cells have evolved several precautionary mechanisms. One of these involves the activity of special DNA polymerases—known as translesion synthesis (TLS) polymerases. In contrast to the replicative polymerases responsible for faithfully duplicating the genome, these can carry out DNA synthesis even on a damaged template. For that to occur, they have to take over synthesis from the replicative polymerase that is stalled at a DNA lesion. Although this mechanism allows DNA synthesis to proceed, TLS polymerases work with a high error rate even on undamaged DNA, leading to alterations of the original sequence that can result in cancer. Consequently, the exchange between replicative and special polymerases has to be highly regulated, and the details of this are largely unknown. Here we identified Def1—a protein involved in the degradation of RNA polymerase II—as a prerequisite for error-prone DNA synthesis in yeast. We showed that after treating the cells with a DNA damaging agent, Def1 promoted the degradation of the catalytic subunit of the replicative DNA polymerase δ, without affecting the other two subunits of the polymerase. Our data suggest that the special polymerases can take over synthesis only after the catalytic subunit of the replicative polymerase is removed from the stalled fork in a regulated manner. We predict that the other two subunits remain at the fork and participate in TLS together with the special polymerases.

## Introduction

The stalling of the replication machinery that occurs as a consequence of encountering unrepaired DNA damages is a challenging problem for cells. Stalled replication forks can undergo DNA breakage and recombination that can lead to chromosomal rearrangements and cell death. To ensure survival, cells have evolved different mechanisms that can sustain DNA replication on damaged templates. These so-called DNA damage tolerance or DNA damage bypass processes allow replication to continue on damaged DNA without actually removing the damage. DNA damage tolerance is achieved through two main mechanisms: template switching and translesion synthesis (TLS). Template switching is inherently error-free, as replication continues by using the undamaged nascent sister chromatid as a template for the bypass of the lesion [1], whereas during TLS, specialized polymerases take over the nascent primer end from the replicative polymerase and carry out synthesis opposite the DNA lesion in an error-free or error-prone way [2].

Rad6 and Rad18 are key mediators of DNA damage tolerance in the yeast *Saccharomyces cerevisiae* [3],[4]. They govern at least three different pathways for the replication of ultraviolet light (UV)-damaged DNA: (1) Rad5-dependent error-free DNA damage bypass, (2) Rad30-dependent error-free TLS, and (3) Rev3-dependent error-prone TLS. Rad6 is an ubiquitin conjugase [5], and it forms a complex in the cell with Rad18, a RING finger ATP-ase with single-stranded DNA-binding activity [6]. Upon UV-treatment, the Rad6–Rad18 ubiquitin–conjugase–ligase complex monoubiquitylates proliferating cell nuclear antigen (PCNA), the essential processivity factor for replicative DNA polymerases, at its lysine-164 residue at the stalled replication fork [7]. Monoubiquitylated PCNA activates the Rev3, and the Rad30-dependent subpathways involving TLS polymerases, whereas further polyubiquitylation of PCNA on the same residue through a lysine-63–linked chain by the Rad5–Mms2–Ubc13 ubiquitin–conjugase–ligase complex activates the Rad5 subpathway [7],[8]. Genetic experiments suggest that the Rad5 branch operates through template switching, where the newly synthesized strand of the undamaged sister duplex serves as a template to bypass the lesion [9]. Rad5, a SWI–SNF family member helicase, most probably directly promotes this process through its fork-reversal activity [10]. The *RAD30*-encoded DNA polymerase η (Polη) is unique in its ability to efficiently and accurately synthesize through UV-induced cyclobutane pyrimidine dimers [11]. In accordance with its role in the error-free bypass of UV lesions, a defect of Polη in yeast confers an increase in UV-induced mutations, and in humans it causes the cancer-prone syndrome, the variant form of xeroderma pigmentosum [12]–[14]. Besides UV-lesions, Polη can bypass several DNA distorting lesions with varying accuracy [2]. The mutagenic branch involves Rev1 and Rev7, besides Rev3, and the lack of either protein causes immutability [15]. The Rev1 protein is a DNA polymerase with limited ability to insert C residues [16]. Its catalytic activity is dispensable for most induced mutagenesis events, suggesting a mainly structural role for Rev1 [17]. Rev3 together with Rev7 forms DNA polymerase ζ (Polζ) [18]. Rev7 is an accessory protein, whereas Rev3 is the catalytic subunit. Polζ has the ability to efficiently extend from mispaired nucleotides and from nucleotides inserted opposite different DNA lesions [2].

TLS polymerases synthesize DNA with a high error rate and are responsible for introducing mutations into the genome during DNA damage bypass, so their replacement of the replicative polymerase must be tightly regulated. However, our understanding of the polymerase switch at DNA damage sites is elusive. Polη, Rev1, and Polζ were shown to interact with PCNA, and it was suggested that through these interactions TLS polymerases could get access to the replication fork [19]–[21]. Also, the interaction with PCNA was shown to be essential for the *in vivo* function of all three polymerases. Though PCNA binding can give access to TLS polymerases to the replication fork, the mechanism that allows them to actually take over DNA synthesis from the replicative polymerase during DNA lesion bypass is still unknown.

In this study, we identify Def1 as an indispensable regulator of induced mutagenesis. We show that Def1 promotes the ubiquitylation and subsequent proteasomal degradation of the catalytic subunit of the replicative polymerase after DNA damage treatment. We demonstrate that the noncatalytic subunits of the replicative polymerase are not affected by UV-induced degradation and that they can form a complex with the TLS polymerase Rev1. Based on our results we propose a new model for polymerase exchange at stalled replication forks.

## Results

### Rad5 Forms a Stable Complex with Def1 upon MMS Treatment

**Figure 1:**
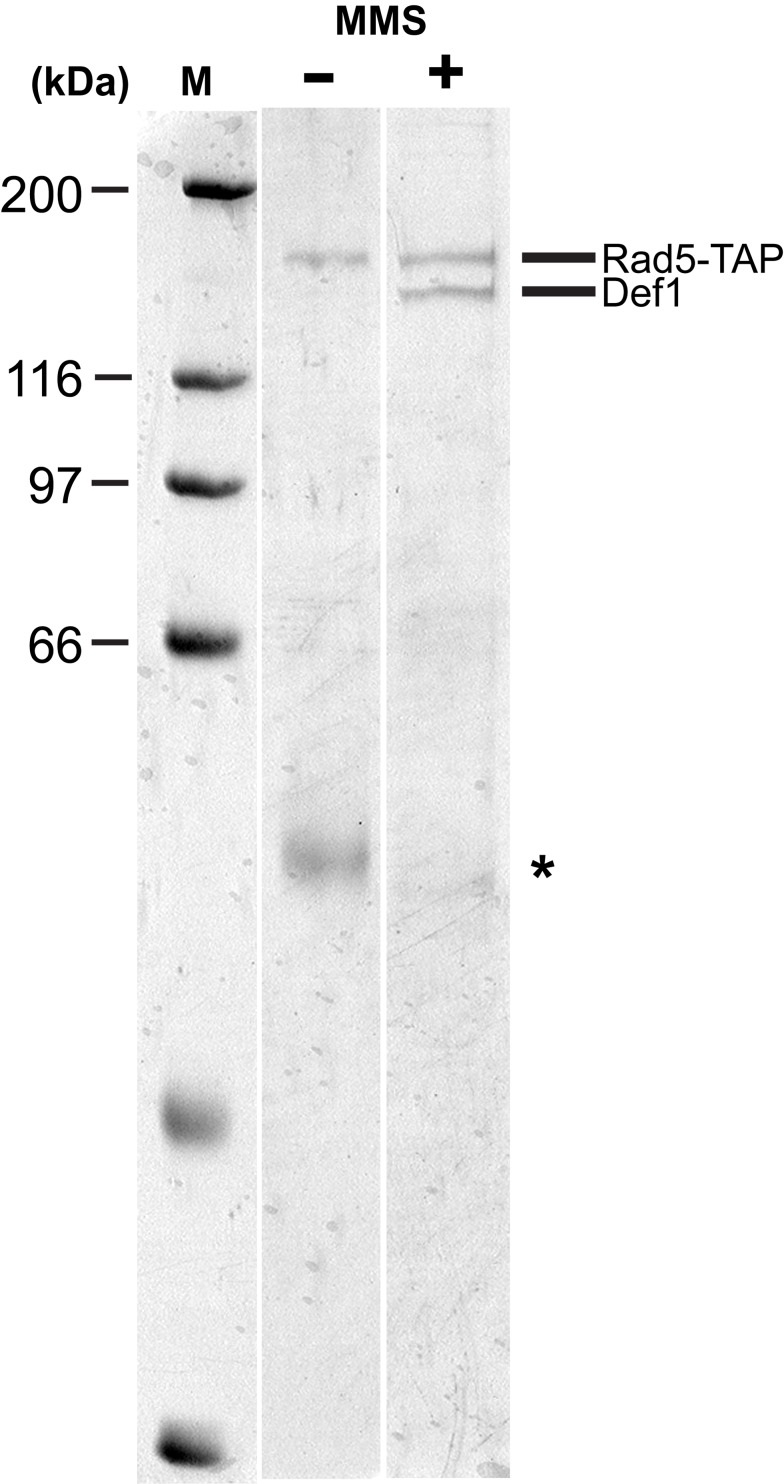
TAP of Rad5 and its complexes. The final elution fractions of TAP purification of whole cell extracts from control and MMS-treated cultures were analyzed by SDS PAGE. Purified proteins are designated on the right, and the molecular mass markers are indicated on the left. The asterisk denotes a nonspecific band.

In searching for new factors affecting DNA damage tolerance, we aimed to identify new interacting partners of Rad5. Therefore, we performed tandem affinity purification (TAP) of Rad5 together with its complexes. For that purpose, we introduced a TAP tag, consisting of a calmoduline binding peptide and two IgG binding domains of protein A separated by a TEV protease cleavage site, at the C-terminus of Rad5 at the chromosomal locus. We purified Rad5 and its interacting partners through the two affinity tags under native conditions. To facilitate the formation of damage bypass complexes, we applied 0.02% methyl methanesulfonate (MMS) for 2 h before collecting the cells. Surprisingly, without treatment only one prominent, specific band was visible in the final, highly purified fraction on the Coomassie-stained gel (Figure 1), which was identified by mass spectrometry as the tagged Rad5 itself. However, after MMS treatment two prominent bands appeared on the gel; the lower mobility band was again Rad5, whereas the higher mobility band was identified as Def1. Repetition of the experiment yielded the same result that Def1 copurified with Rad5, but only after treating the cells with MMS. It suggested that either DNA-damage–induced posttranslational modification of Rad5 and/or Def1 or a third, damage-specific factor was necessary to promote the formation of the complex.

### *DEF1* Functions in the *RAD6*-Mediated DNA Damage Tolerance

**Figure 2:**
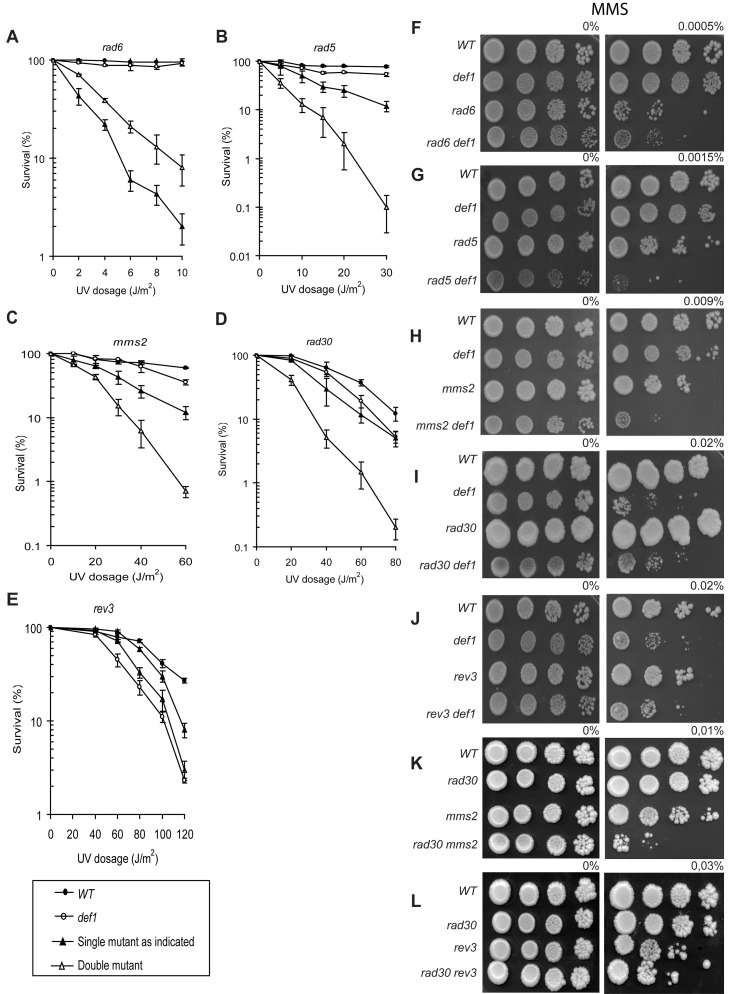
*DEF1* participates in the *REV3* branch of the *RAD6*-governed DNA damage tolerance. (A–E) Epistatic analysis of *DEF1* with mutants of the different branches of the *RAD6* pathway upon UV irradiation. Standard deviations are indicated. (F–J) Epistatic analysis of the same mutants upon MMS treatment. (K, L) Genetic interactions of *RAD30* with *MMS2* and *REV3* upon MMS treatment. All experiments were repeated at least three times.

Rad5 mediates an error-free DNA damage tolerance pathway under the control of Rad6–Rad18, whereas Def1 has a role in promoting the proteolytic degradation of stalled RNA polymerase II and in telomere maintenance [22],[23]. To further establish a connection between Rad5 and Def1, we analyzed the genetic relations between *DEF1* and *RAD5*, and members of all three branches of the *RAD6*-governed pathway upon DNA damage. After treating the cells with UV, the sensitivity of the *def1 rad6* double deletion strain did not exceed that of the *rad6* mutant, pointing to an epistatic relationship between *DEF1* and *RAD6* (Figure 2A). The higher resistance seen with the double mutant might originate from other functions of these multitask proteins, as the *def1 rad18* strain showed the same sensitivity as *rad18* (unpublished data), fortifying the involvement of *DEF1* in the *RAD6-*dependent DNA damage tolerance. Surprisingly, the *def1 rad5* double deletion strain displayed much higher sensitivity than any of the corresponding single mutants, indicating that *DEF1* acted outside of the *RAD5*-dependent subpathway (Figures 2B). This was verified by the hypersensitivity of the *def1 mms2* strain over the single mutants (Figure 2C). Also, the *def1 rad30* double mutant was more sensitive to UV than either *def1* or *rad30*, implying that *DEF1* functioned independently of *RAD30* (Figure 2D). Nevertheless, the *def1 rev3* strain exhibited the same sensitivity as the *def1* mutant, which indicated an epistatic relationship between *DEF1* and *REV3* (Figure 2E). We carried out similar experiments using MMS instead of UV as a DNA damage source (Figures 2F–J). Upon MMS treatment *DEF1* showed epistasis with *RAD6* and *REV3*, but its deletion further sensitised *rad5* and *mms2*, proving again the involvement of *DEF1* in the mutagenic branch of the *RAD6*-governed DNA damage tolerance. However, *DEF1* also showed epistasis with *RAD30*, as the double mutant was as sensitive as the *def1* single mutant. We note that this reflected a real epistatic relationship, as although *rad30* itself was not sensitive to MMS, only at very high doses, it was hypersensitive with *mms2*, but also showed epistasis with *REV3* (Figure 2K,L). That means that in the bypass of MMS-induced DNA lesions, *RAD30* works together with the members of the *REV3* branch. In conclusion, our data strongly suggested that *DEF1* participated in the *REV3*-dependent mutagenic branch of the *RAD6*–*RAD18*-regulated DNA damage tolerance.

### DNA-Damage–Induced Mutagenesis Is Abolished in *def1* Deletion Mutants

**Figure 3:**
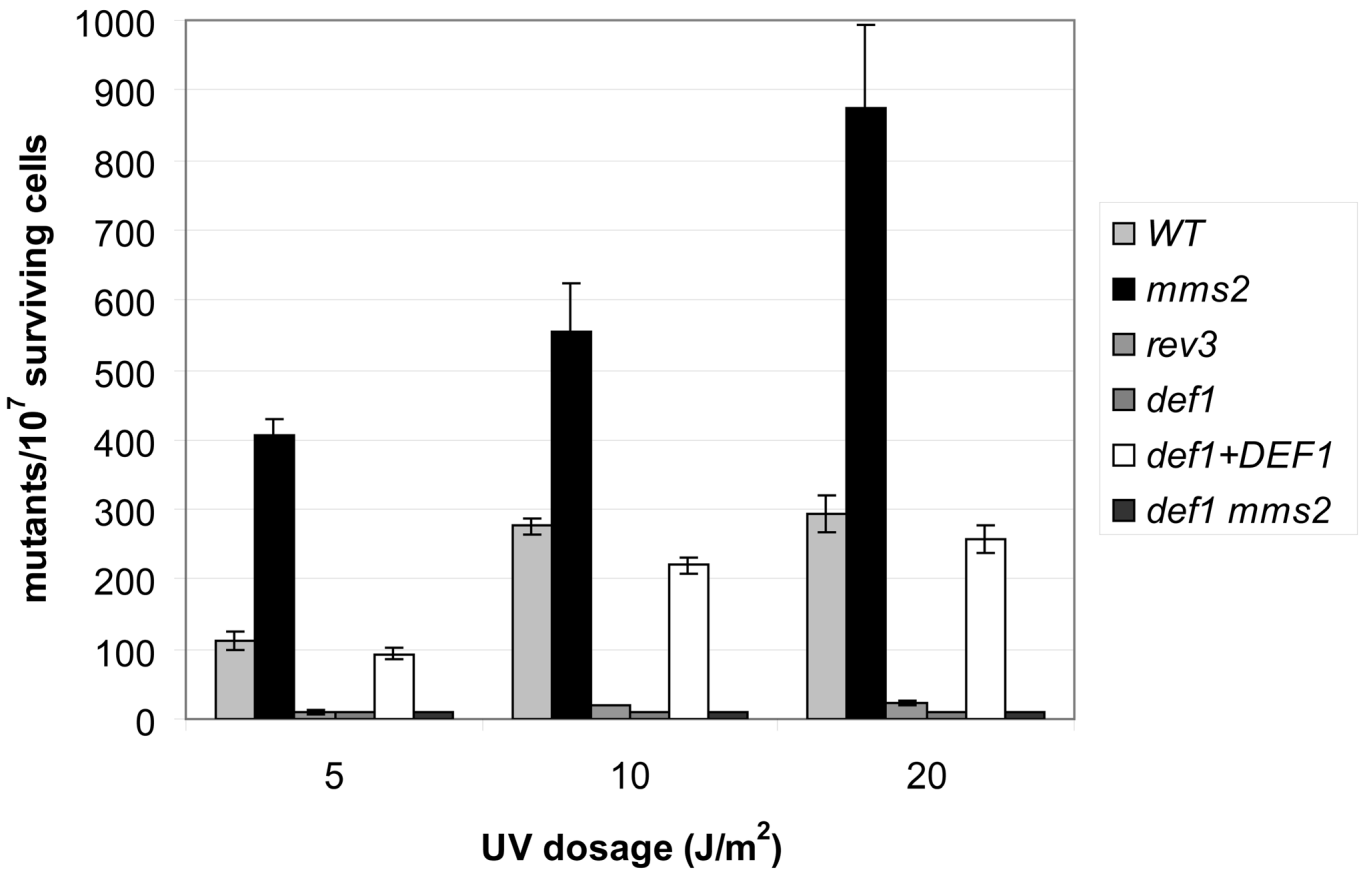
DNA-damage–induced mutagenesis is abolished in *def1* deletion mutants. Forward mutation rates at the *CAN1* locus were determined after UV treatment. Where indicated, *def1* deletion was complemented by wild-type *DEF1* expressed under the control of the ADH1 promoter on a centromeric plasmid. The standard deviation is indicated above each bar. Experiments were repeated three times.

The TLS polymerases of the *REV3* branch are responsible for virtually all damage-induced mutagenesis; consequently, inactivation of either one causes a strong antimutator effect [15]. To prove that *DEF1* belonged to the *REV3* branch, we measured the rate of UV-induced mutations in *def1* strains. In keeping with the results of the epistasis analysis, induced mutagenesis was completely abolished in *def1* (Figure 3). In fact, *def1* was even more defective than the *rev3* strain. Additional deletion of *DEF1* in *mms2* also eliminated induced mutagenesis, though *mms2* by itself causes high mutagenesis, most probably because in the absence of the error-free branch, lesions are channelled to the *REV3*-dependent mutagenic pathway. Ectopic expression of Def1 in *def1* cells restored close to wild-type–level mutagenesis, confirming that the immutability was in fact due to the absence of *DEF1*. We obtained the same results using MMS instead of UV (unpublished data). From these we concluded that *DEF1* played an essential role in induced mutagenesis.

### Pol3 Is Degraded upon DNA Damage by a Def1-Dependent Manner

**Figure 4:**
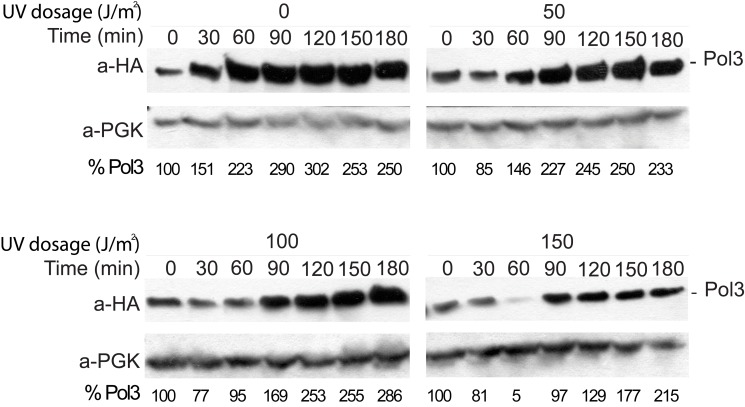
UV-dose–dependent degradation of Pol3. Cultures of *mms2* cells were synchronized by α-factor and irradiated with increasing doses of UV, as indicated. After released back to growth, 1 ml of cells was collected at the indicated time points, and cell extracts were analyzed by Western blotting. Anti-HA detected HA-tagged Pol3, and PGK served as a loading control. The level of Pol3 relative to PGK is shown at the bottom of each panel.

**Figure 5:**
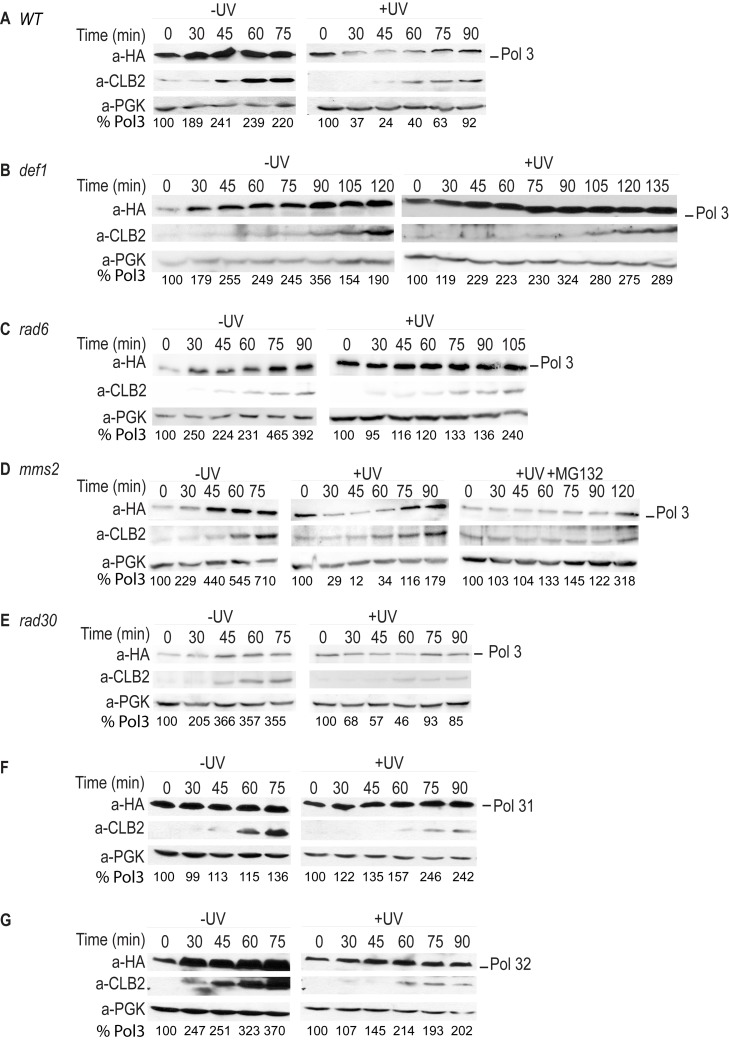
Pol3 degradation depends on *RAD6* and *DEF1*. Cultures were synchronized by α-factor, UV-irradiated with 150 J/m^2^, and released back to growth media. Proteins from whole cell extracts, prepared from 1 ml of cell culture collected at the indicated time points after UV treatment, were analyzed by Western blotting. Anti-HA antibody was used to detect HA-tagged Pol3 (A to E), Pol31 (F), or Pol32 (G). Cell cycle progression was monitored by Clb2 cyclin levels, and PGK served as a loading control. The level of Pol3 relative to PGK is shown at the bottom of each panel.

For the *REV3* branch to operate, the TLS polymerases of the branch have to take over synthesis from the replicative polymerase stalled at a DNA lesion site, a central but poorly understood step in DNA lesion bypass. Because Def1, unlike other members of the *REV3* branch, is not a DNA polymerase, we surmised that it might facilitate the exchange between the TLS and the replicative polymerases. As Def1 played a role in the ubiquitylation of stalled RNA polymerase II [22], we considered the possibility that, similarly, it could mediate ubiquitylation of the stalled replicative DNA polymerase. Ubiquitylation then could lead to polymerase switch by either playing a regulatory role as in the case of DNA-damage–induced ubiquitylation of PCNA [7], or it could result in protein removal through degradation. To test these possibilities, we followed the fate of the replicative polymerase during DNA damage bypass by monitoring Pol3, the catalytic subunit of the replicative DNA polymerase δ (Polδ) during cell cycle in UV-treated, synchronized yeast cultures. In order to facilitate TLS, we first used an *mms2* deletion strain. Importantly, we observed a transient decrease in the level of Pol3 upon UV irradiation as opposed to normal growth conditions, and the degree of degradation correlated with the applied UV doses (Figure 4). We could also detect degradation in wild-type cells in the S phase of the cell cycle, as indicated by the expression pattern of the G2/M-specific cyclin Clb2 (Figure 5A). However, in experiments using a *def1* deletion strain, we could not detect any decrease in the level of Pol3 (Figure 5B). To investigate whether the observed phenomenon was ultimately under the higher control of *RAD6*, we performed the same experiment in a *rad6* strain and found that Pol3 diminution was also absent (Figure 5C). On the other hand, reduction of Pol3 could be seen in *mms2* (Figure 5D) and in *rad30* (Figure 5E) backgrounds. These results, in conjunction with the above genetic results, strongly implied that Pol3 diminution was specifically dependent on *DEF1*, which exerted this function under the control of *RAD6*, in the mutagenic DNA damage bypass pathway.

### Def1 Induces the Ubiquitylation and Proteasomal Degradation of Pol3

**Figure 6:**
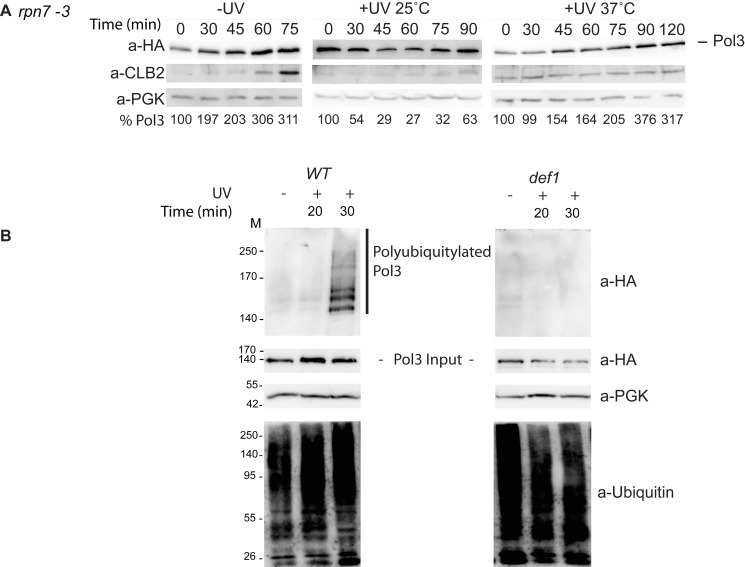
UV-induced degradation of Pol3 is mediated by the proteasome and is triggered by its Def1-dependent polyubiquitylation. (A) The *rpn7-3* mutant is deficient in degrading Pol3. Experiments were done as described in Figure 5, except that for the inactivation of the proteasome, cells were shifted to 37°C 2 h before α factor treatment, and after 3 h of synchronization, irradiated with 200 J/m^2^. Anti-HA antibody was used to detect HA-tagged Pol3. Cell cycle progression was monitored by Clb2 cyclin levels, and PGK served as a loading control. The level of Pol3 relative to PGK is shown at the bottom of each panel. (B) Def1 assists Pol3 polyubiquitylation. Polyubiquitylated proteins from cell extracts prepared from 100 ml of cell culture before and 20 and 30 min after UV irradiation were bound to NiNTA agarose (Qiagen), and Pol3 was identified in the bound fraction with HA antibody (upper panels). The PGK and Pol3 levels in the extracts before adding to the beads are also shown, and the bound fractions were probed with anti-ubiquitin antibody (lower panels). The applied UV dose was 150 J/m^2^.

The most plausible explanation for the transient decrease of Pol3 would be that Pol3 underwent regulated protein degradation induced by UV. The majority of regulated proteolysis takes place in the proteasome in eukaryotic cells. To resolve whether the decrease in the Pol3 protein level was due to protein degradation mediated by the proteasome, we supplemented the growth media with the proteasome inhibitor MG132. Indeed, in the presence of MG132, the UV-induced degradation of Pol3 could not be observed (Figure 5D). To add further evidence, we applied a temperature-sensitive *rpn7* mutant displaying defects in proteasome function at high temperature (37°C) but behaving like wild-type at low temperature (25°C) [24]. Using this mutant we could not detect degradation at the restrictive high temperature contrary to the permissive low temperature (Figure 6A), whereas in the *RP* *N* *7* strain degradation occurred at both temperatures (unpublished data). These results demonstrated that the proteasome was responsible for the UV-induced degradation of Pol3.

**Figure 7:**
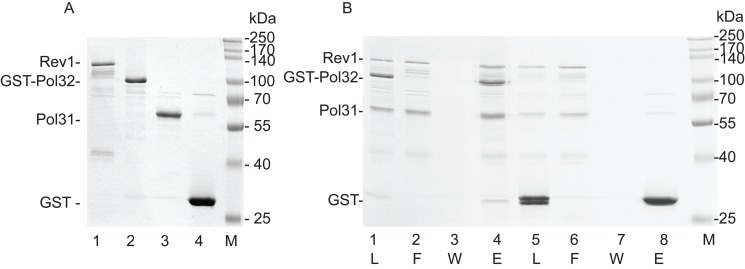
Rev1 forms a complex with Pol31 and Pol32. (A) Purity of the proteins. We analyzed 0.5 µg of each protein on a 10% denaturing SDS-polyacrylamide gel. Molecular mass standards are shown on the right. (B) GST pull-down of the purified proteins. GST–Pol32 immobilized on glutathione–Sepharose beads was incubated with purified Pol31 and Rev1. After washing, bound proteins were eluted with glutathione. Aliquots of each sample, taken before addition to the beads (L), from the flow-through fraction (F), from the last wash (W), and from the glutathione-eluted proteins (E), were analyzed on 10% SDS-polyacrylamide gel (lanes 1–4). The results for the control experiment using GST instead of GST–Pol32 are shown in lanes 5–8. Molecular mass standards are shown on the right.

Ubiquitylation is a major signal for proteasomal protein degradation. To show ubiquitylation of Pol3, N-terminally 7 histidine-tagged ubiquitin was expressed in yeast cells and ubiquitylated proteins from cell extracts prepared after irradiating cells with UV were enriched on nickel beads. Indeed, we could detect polyubiquitylated forms of Pol3 upon UV irradiation in wild-type cells, but not in *def1* and *rad6* cells (Figure 7B and unpublished data).

### Pol31 and Pol32 Are Not Subject to UV-Induced Degradation

Polδ is a heterotrimer and consists of two noncatalytic subunits, Pol31 and Pol32, besides Pol3 [25]. Pol31, like Pol3, is essential for cell viability, but Pol32 is a nonessential subunit. Pol3 forms a stable complex with Pol31, and Pol32 is attached to this complex through its interaction with Pol31 [26]. We aimed to examine whether the whole Polδ enzyme was subject to UV-induced proteolysis, or it affected only the catalytic subunit. We found that contrary to Pol3, Pol31 and Pol32 were not affected by UV-induced degradation (Figure 5F and 5G).

### Pol31 and Pol32 Can Form a Complex with Rev1

Taken together, these results suggested that during DNA damage bypass, Pol31 and Pol32 remained at the stalled fork. We postulated that a TLS polymerase could take the place of Pol3 and carry out lesion bypass in complex with Pol31 and Pol32. To test this idea, we examined whether Pol31 and Pol32 together could form a complex with Rev1 in *in vitro* assays using purified proteins. We chose Rev1, because it had been suggested to function as a scaffold in TLS, based on its interaction in yeast with Polη and Polζ [27],[28], and in mouse and human cells with Polη, Polι, and Polκ [29],[30]. Also, it has already been shown to interact with Pol32 [31]. In GST pull-down assays we added Pol31 and Rev1 to GST–Pol32 immobilised on glutathione–Sepharose affinity beads, and after incubation bound proteins were released from the beads by glutathione. As shown in Figure 7B lanes 1–4, both Pol31 and Rev1 eluted together with GST–Pol32, indicating that these proteins formed a complex together. In control experiments using GST instead of GST–Pol32, only GST was present in the elution fraction, confirming that the interaction between Pol31, Pol32, and Rev1 was specific (Figure 7B, lanes 5–8). In conclusion, purified Pol31, Pol32, and Rev1 could interact directly and form a stable multisubunit protein complex.

## Discussion

**Figure 8:**
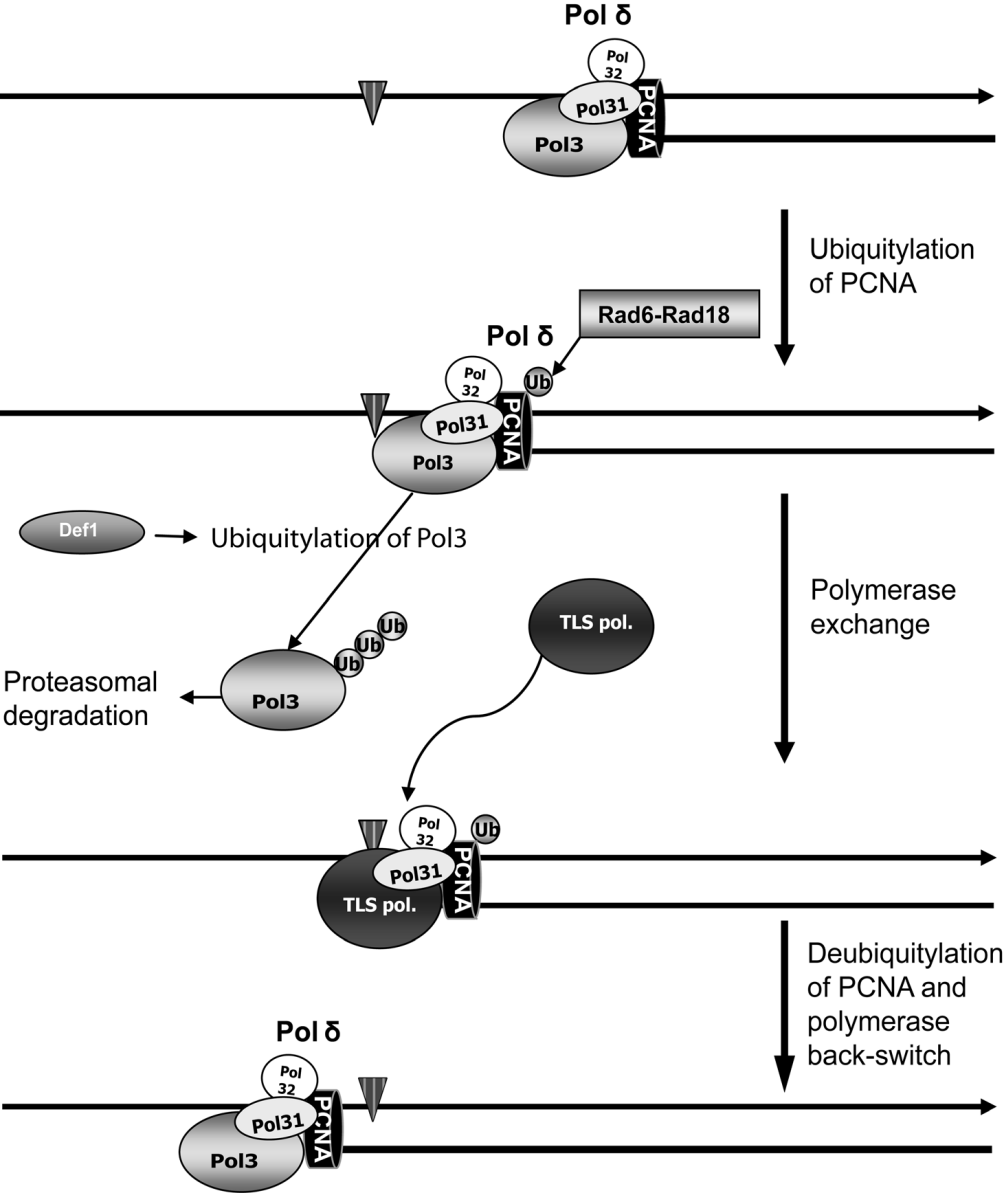
Model for polymerase exchange at a DNA damage site. DNA damage stalls the replication complex and triggers the ubiquitylation of PCNA by Rad6–Rad18 at the stalled fork. Monoubiquitylated PCNA promotes TLS, for which to occur first Pol3 is removed from the stalled complex through ubiquitylation-mediated proteasomal degradation, assisted by Def1. A TLS polymerase takes over the place of Pol3, and together with Pol31 and Pol32 carries out lesion bypass. After the deubiquitylation of PCNA, Pol3 regains its place at the replication complex, and normal replication resumes. For simplicity, only half of the replication fork is shown. The DNA damage site on the template strand is marked by a black diamond symbol.

In this study we identified a DNA-damage–induced complex of Rad5 with Def1. Our genetic studies placed *DEF1* in the *RAD6*–*RAD18*-dependent DNA damage tolerance pathway, where it played an indispensible role during induced mutagenesis. We established that Pol3, the catalytic subunit of the replicative DNA polymerase Polδ, was degraded upon UV irradiation. We presented evidence that degradation of Pol3 was the result of polyubiqitylation-mediated proteasomal degradation, and it was dependent on *DEF1* under the higher control of *RAD6*. Conversely, Pol31 and Pol32, the other two subunits of Polδ, were not degraded. We also demonstrated that Pol31 and Pol32 together could form a stable complex with the TLS polymerase Rev1. Based on these results, we propose a new model for polymerase exchange at stalled replication forks (Figure 8). During replication, when Polδ stalls at a DNA lesion, PCNA gets ubiquitylated by Rad6–Rad18. Monoubiquitylated PCNA activates TLS, for which to occur Pol3 is ubiquitylated by a Def1-dependent manner and removed from the stalled Polδ complex through proteasomal degradation. We hypothesize that a TLS polymerase takes over the place of Pol3 and teams up with the remaining Polδ subunits, Pol31 and Pol32, at the stalled fork to form a new complex capable of executing DNA lesion bypass, suggested by our results showing complex formation of Rev1–Pol31–Pol32, and also by recent studies detecting complex formation of yeast Pol31–Pol32–Rev3–Rev7 and also of their human counterparts [32]–[34]. We surmise that after lesion bypass and deubiquitylation of PCNA, the TLS polymerase is removed from the primer terminus, Pol3 restores Polδ by regaining its place, and replication continues. Importantly, this model gives an explanation for previous genetic results showing that in *pol32* cells induced mutagenesis is severely impaired, and that deletion of the N-terminal part of Pol32, responsible for binding Pol31, also abolishes induced mutagenesis [25],[35],[36].

Our data raise an important question: How can the *RAD30*-encoded TLS polymerase, Polη, operate independently of Def1? Our results imply that Pol3 does not have to be removed from the stalled fork for Polη-dependent UV-lesion bypass to occur. Polη is mainly specialized for the error-free bypass of UV lesions, so it is reasonable to assume that Polη should have preference over the error-prone TLS polymerases in the bypass of UV-induced DNA damages. Polη differs from the other TLS polymerases, Rev1 and Polζ, in its way of binding PCNA. Although Rev1 and Polζ bind the intermolecular interface at the outer face of the PCNA ring [20],[21], Polη, similarly to Polδ, binds the interdomain connector loop of PCNA through its conserved PCNA-interacting peptide motif [19]. Given that PCNA is a homotrimer ring, Polδ and Polη could bind the same PCNA ring simultaneously. We presume that transient conformational changes, probably induced by the stalling of the fork and ubiquitylation of PCNA, could allow Polη to take over synthesis from Polδ, as also suggested by *in vitro* experiments [37], while both remain attached to PCNA. Because Polη synthesizes opposite pyrimidine dimers with the same kinetics as it does opposite undamaged DNA [38], rapid bypass can occur. Deubiquitylation of PCNA would restore the original conformation and Polδ could continue synthesis. We note that this is in accord with the *in vivo* finding that Pol32 is not needed for TT dimer bypass carried out by Polη [39]. On the other hand, when the damage poses a kinetic barrier also to the TLS polymerase, for the slower kinetic damage bypass to occur, Pol3 has to be removed so that the TLS polymerases could form a stable complex with Pol31 and Pol32. This would also explain the epistasis of *RAD30* with *DEF1* in the bypass of MMS-induced DNA lesions, as the efficiency of incorporation by Polη is reduced ∼20-fold opposite ^6^O-methylguanine and ∼1,000-fold opposite an abasic site [40],[41].

We detected a very stable DNA-damage–induced complex formation of Rad5 with Def1. However, our genetic data placed the two genes into two alternative DNA damage tolerance pathways, both governed by Rad6. We hypothesize that the Def1–Rad5 complex might coordinate the activity of the two subpathways in response to DNA damage. A similar role of Def1 during transcription was suggested, where Def1 assisted in the degradation of the RNA polymerase stalled at DNA damage sites, and probably coordinated the repair mechanisms through its interaction with Rad26 [22].

The high conservation between elements of DNA lesion bypass from yeasts to humans, including the Rad6–Rad18 and Rad5–Mms2–Ubc13 complexes and their enzymatic activities, the TLS polymerases, and PCNA ubiquitylation [42], suggests that DNA-damage–induced selective degradation of the catalytic subunit of the replicative DNA polymerase drives polymerase exchange in higher eukaryotes as well. The role of TLS polymerases in mutagenesis and in cancer makes it highly important to uncover further details of polymerase exchange, to identify and investigate further factors that affect Pol3 degradation, and to check the existence of a similar mechanism in human cells.

## Materials and Methods

### Yeast Strains and Plasmids

The wild-type strain (BY4741) and its single deletion derivatives for the genetic studies were obtained from the Euroscarf collection. Chromosomally C-terminally tagged *POL3*, *POL31*, and *POL32* with three copies of the hemagglutinin epitope tag (3-HA) were created by a PCR-based strategy [43] in EMY74.7 (*MAT*a, *his3-*Δ*1*, *leu2–3,–112*, *trp1*Δ, *ura3–52*) strain, made *bar1*Δ. Additional deletions were generated by gene replacement. *RAD5* was TAP tagged in BJ5464 by the same PCR-based strategy using pBS1539 [44]. BJ5464 was also used for protein overexpression. The *rpn7-3* mutant and its corresponding W303 wild-type strain [24] were used in experiments showing the effect of temperature-sensitive inhibition of the proteasome. Polyubiquitylation of Pol3 was shown in MHY500 strain background [45]. For complementation in yeast, Def1 was expressed from the centromeric vector pID394 (p416ADH backbone [46]). For protein purification, Pol31, Pol32, and Rev1 were overexpressed in N-terminal GST fusion from pID370, pID458, and pID460, respectively (pBJ842 backbone [47]). In the plasmid pRS426–pCUP1–His7–Ubiquitin (G76A) [48], the mutation was reversed by site-directed mutagenesis, resulting in plasmid pID198.

### Sensitivity Assays

For qualitative analysis of sensitivity to MMS, cells were serial diluted and spotted onto YPD plates containing defined amounts of MMS and grown at 30°C for 3–5 d. For quantification, cells were spread onto YPD plates at appropriate dilutions and irradiated with UV light (254 nm) for varying times to apply the specified dosage. Plates were incubated in the dark at 30°C, and colonies were counted after 3–5 d.

### UV-Induced Mutation Rate

UV-induced forward mutation frequencies at the *CAN1* locus were measured by comparing the numbers of *can1^R^* colonies at given UV doses, selected on synthetic complete medium without arginine and containing canavanine, with the numbers of survivors on complete synthetic medium, exposed to the same UV doses.

### Cell Synchronisation

Logarithmically growing cells in YPD at 30°C were arrested at A_600_∶0.8 in G1 by 100 ng/µl α-factor (Sigma) for 2–4 h, washed, resuspended in phosphate buffered saline, and divided into Petri dishes for UV irradiation. Half of the cultures were irradiated with the given UV dose, and the other half served as untreated control. Cells were released back into growth medium containing 50 µg/ml pronase (Calbiochem) to inactivate any residual α-factor. For experiments showing polyubiquitylation of Pol3, the growth media always contained 100 µM CuSO_4_ to induce 7His–ubiquitin expression. Samples were taken at given time points after UV treatment for whole cell extract preparation. Experiments involving MG132 (Sigma) were done in *pdr5* background. MG132 (50 µM) was added to the α-factor synchronized cultures 1 h before UV irradiation. The *rpn7-3* mutant and its isogenic wild-type strain were grown at 25°C. To detect the mutant phenotype we followed the protocol described in [24]. Briefly, 50 ml culture of logarithmically growing cells (A_600_∶0.5) were split. Half of the culture was kept at 25°C and the other half was shifted to 37°C. At A_600_∶0.8 cultures were synchronised by α-factor for 3 h and processed as detailed above.

### Protein Techniques and Antibodies

Whole cell extracts were prepared according to a trichloroacetic acid (TCA) protein precipitation method [43] except that after TCA precipitation, pellets were washed with ice-cold acetone, air-dried, and resuspended in 1× Laemmli sample buffer before loading to an 8% poly-acrylamide gel. Polyubiquitylated Pol3 was detected using denaturing NiNTA chromatography as described in [49]. Antibodies against HA (Gene Tex), Clb2 (Santa Cruz), PGK (Molecular Probes), and ubiquitin (Santa Cruz) were used. Pol31, Pol32, and Rev1 were overexpressed in N-terminal fusion with GST and purified on glutathione–Sepharose 4B beads following the protocol in [45], with the exception that in the case of Rev1, 0.1% Triton X-100 was added to the lysate after breaking the cells. In the case of Pol31 and Rev1, the GST tag was removed by PreScission protease cleavage in the elution step of purification. For complex formation, GST–Pol32 (3 µg) immobilized on glutathione–Sepharose beads was incubated with purified Pol31 (5 µg) and Rev1 (3 µg), overnight on ice in buffer containing 50 mM Tris/HCl, pH 7.5, 150 mM NaCl, 1 mM EDTA, 1 mM DTT, 10% glycerol, 0.01% Nonidet P-40. Beads were washed five times with the same buffer, and bound proteins were eluted in the same buffer containing 20 mM reduced glutathione. Various fractions were analyzed by SDS/PAGE.

### TAP

Four liters of yeast culture were grown to logarithmic phase in synthetic complete medium, and at A_600_∶0.8, half of the culture was treated with 0.02% MMS for 2 h before harvesting. TAP purification was carried out as described [44] with the following modifications: cells were broken in 1× YBB (50 mM Tris/HCl pH:7.5, 50 mM KCl, 100 mM NaCl, 10% sucrose) supplemented with protease inhibitors. After clarifying the lysate with ultracentrifugation for 1 h with 100,000 g, 2-mercaptoethanol was added to 8.5 mM, Nonidet P-40 to 0.01%, and NaCl to 500 mM final concentration, and the lysate was transferred into an IgG Sepharose bead (Amersham)–filled column. In later steps the protocol was followed. Briefly, bound fraction was eluted with TEV protease cleavage. The elution fraction was applied on calmodulin beads, and bound proteins were recovered by eluting in EGTA-containing buffer. Proteins were concentrated and analysed on a 6%–12% gradient sodium dodecyl sulphate polyacrylamide gel stained with Coomassie blue R-250. Excised protein bands were identified by MALDI-TOF mass spectrometry after trypsin digestion. Eleven peptides of the higher mobility band matched yeast Def1 (55% coverage) and they covered 18% of the *DEF1* sequence.

## Abbreviations

GST: glutathione S-transferase
HA: hemagglutinin
MMS: methyl methanesulfonate
PCNA: proliferating cell nuclear antigen
Pol: polymerase
TAP: tandem affinity purification
TLS: translesion synthesis
UV: ultraviolet light
